# Influence of Accelerators on Cement Mortars Using Fluid Catalytic Cracking Catalyst Residue (FCC): Enhanced Mechanical Properties at Early Curing Ages

**DOI:** 10.3390/ma17051219

**Published:** 2024-03-06

**Authors:** Lourdes Soriano, María Victoria Borrachero, Ester Giménez-Carbo, Mauro M. Tashima, José María Monzó, Jordi Payá

**Affiliations:** Giquima—Research Group of Building Materials Chemistry, Instituto Universitario de Investigación de Ciencia y Tecnología del Hormigón ICITECH, Universitat Politècnica de València, 46022 Valencia, Spain; vborrachero@cst.upv.es (M.V.B.); esgimen@cst.upv.es (E.G.-C.); maumitta@upvnet.upv.es (M.M.T.); jmmonzo@cst.upv.es (J.M.M.); jjpaya@cst.upv.es (J.P.)

**Keywords:** pozzolan, accelerator, compressive strength, thermogravimetry

## Abstract

Supplementary cementitious materials (SCMs) have been used in the construction industry to mainly reduce the greenhouse gas emissions associated with Portland cement. Of SCMs, the petrochemical industry waste known as fluid catalytic cracking catalyst residue (FCC) is recognized for its high reactivity. Nevertheless, the binders produced using SCMs usually present low mechanical strength at early curing ages. This study aims to assess the effect of different accelerating additives (KOH, sodium silicate SIL, commercial additive SKR) on the mechanical strength of mortars containing FCC. The results show that after only 8 curing hours, the compressive strength gain of the FCC mortars containing SKR was over 100% compared to the FCC mortar with no additive (26.0 vs. 12.8 MPa). Comparing the compressive strength of FCC mortar containing SKR to the control mortar, the enhancement is spetacular (6.85 vs. 26.03 MPa). The effectiveness of the tested accelerators at 8–24 curing hours was KOH ≈ SIL < SKR, whereas it was KOH < SIL < SKR for 48 h–28 days. The thermogravimetric data confirmed the good compatibility of FCC and the commercial accelerator.

## 1. Introduction

The construction industry is responsible for consuming significant amounts of raw materials, and Portland cement (PC) manufacturing produces around 7–8% of anthropogenic CO_2_ [1]. For decades, studies into supplementary cementitious materials (SCMs) have demonstrated their viability as partial replacements of PC, and for providing technological and environmental benefits [2]. The most widely used SCMs are fly ash (FA) [3,4,5], silica fume (SF) [6,7], or blast furnace slag (BFS) [8,9]. Fluid catalytic cracking catalyst residue (FCC) use has been studied since the late 1990s by various research groups worldwide [10,11,12,13]. In the first research work into this waste, Payá et al. [12] have demonstrated the importance of previous FCC grinding to enhance pozzolanic reactivity. Optimal cement FCC substitution percentages fall within the 15–20% range, and performance is enhanced when using FCC by replacing sand in mortars’ dose [12]. Pacewska et al. [14] have compared employing FCC with SF and FA in paste. They concluded that the pastes with FCC reached double the compressive strength of the control, and that FCC performed better than the other two tested pozzolans. Lu et al. [13] have recently produced high-performance, high-speed 3D concrete printing (3DCP) using FCC. They stated that FCC use contributes to reducing CO_2_ emissions by up to 21.45% and costs by about 18% compared to the control concrete. FCC was utilized as pozzolan in the construction of a footbridge over the Ovejas Ravine in Alicante (Spain) [15]. The footbridge was made with ultra-high-performance fiber-reinforced concrete that contained a high percentage of FCC.

In recent years, the construction world has faced formidable challenges due to the high demand of natural resources needed for manufacturing, emissions from using cement, and shortages of skilled labor, among other problems. The use of precast or prefabricated concrete offers advantages over traditional concrete constructions. Other advantages include better quality controls and cost reductions [16,17]. In addition to good performance for long curing times, the production of prefabricated specimens requires high compressive strength at early curing ages. This technological demand is usually associated with thermal curing [18] and/or using hydration accelerating additives [19].

As established by Tao et al. [20], accelerators can be classified as follows: (a) effect (enhancing setting or hardening); (b) composition (organic or inorganic); (c) type of application or action (physical or chemical action). A setting accelerator affects mainly the C_3_A phase, while a hardening accelerator affects the C_3_S phase. The commonest inorganic salts used as accelerators are alkaline salts of chlorides, nitrates, aluminates, silicates, thiocyanates, nitrates, carbonates, and alkaline hydroxides. The most frequently employed organic salts used as accelerators are alkanolamines, as well as carboxylic and hydrocarboxylic acids and their salts [20]. In the literature, many research works have used accelerator additives to enhance mechanical properties in the first curing hours [21,22,23]. Ananyachandran and Vasugi [24] employed calcium nitrate and triethanolamine in mortars with metakaolin (MK) and BFS to study the effects of additives on mortars with pozzolans. After 1 curing day, the compressive strength of the mortar with MK was around 30 MPa versus 24 MPa and 20 MPa for the mortar with BFS and the control mortar, respectively. The results demonstrate that accelerators may improve mechanical strength.

Hence, in this study, the effect of accelerator additives (KOH, sodium silicate, and a commercial additive) were tested for mortars in which FCC partially replaced sand at 10 wt.%. These accelerator additives were selected, considering that the most commonly used inorganic additives are inorganic salts containing sodium and calcium combined with alkaline hydroxides and thiosulfates, among others.

Compressive strength was assessed for 8, 24, and 48 h, and for 28 curing days. Thermogravimetric studies were performed in pastes to study the influence of the accelerating additions.

The main scientific contribution of this research is to obtain mortars with high mechanical compressive strength during the first curing ages by combining the use of a highly reactive residual pozzolan and accelerator additives.

## 2. Materials and Methods

The materials employed to fabricate mortars and pastes were cement CEM I-52.5 R [25] supplied by Cemex (Buñol, Spain) and FCC supplied by BP-Oil (Grao de Castellò, Spain). The FCC was ground for 20 min in a Gabrielli Mill-2 equipment (Gabrielli Technology, Calenzano, Italy) using 98 alumina balls. After the grinding process, the mean particle diameter was reduced from 85.34 µm of the original material to 19.73 µm of the ground material. The chemical compositions of both the FCC and cement are shown in Table 1. The composition determination of the cement and FCC was carried out by using a Philips MagiXPRO X-ray fluorescence equipment.

As accelerators, a solution of KOH 0.045 M, sodium silicate (SIL), (64% H_2_O, 28% SiO_2_, 8% Na_2_O), and a commercial accelerator called Sika Rapid-1 (SKR) (Sika Spain, Alcobendas, Spain) were employed. A superplasticizer was used to obtain mortars with an optimum workability value of 110–120 mm (Sika Viscocrete-5980). The fabricated mortars had a cement/sand ratio of 1:3 and a water/cement ratio of 0.45. The employed sand was siliceous in nature and complied with the requirements established in the Spanish standard [26]. FCC replaced 10% of the sand weight, and the quantity of cement remained constant. The mixture method and the compressive strength assay followed the steps indicated in the Spanish standard [26]. The compressive strength test was performed using an Ibertest MUP-60 universal test (Ibertest, Madrid, Spain).

The composition and nomenclature of mortars are listed in Table 2.

To quantify the contribution of pozzolan and the additive to the strength of mortars, the Resistance Activity Index (RAI) was calculated. This value was obtained as the ratio between the strength of each mortar and the control mortar without the addition of accelerator nor mineral addition. The curing ages chosen to perform the compression test were 8, 24, and 48 curing hours to evaluate early curing ages, and 28 days to assess the influence of the accelerator additives on the long-term curing age.

Thermogravimetric studies were carried out on cement pastes with a substitution percentage of 15% cement for FCC. Then, 2% SIL and SKR in relation to the binder (cement or cement + FCC) were added to the pastes. KOH was not used because of the poor results obtained in the mechanical strength study. The paste samples were analyzed for 24, 48, and 72 h, and 28 curing days. For each curing age, a piece of the paste sample was milled, and the hydration process was stopped by adding acetone. The mixture was filtered, and the resulting solid was dried at 65 °C for 30 min. The equipment for the thermogravimetric analysis was an 850 TGA Mettler Toledo module (Metller-Toledo S.A.E, Cornellà del Llobregat, Spain). The heating range was 35–600 °C at a heating rate of 10 °C/min. The experiment was run in an N_2_ atmosphere at a flow rate of 75 mL/min. To achieve a water vapor self-generated atmosphere, an aluminum crucible with a pinhole lid was used [27], which led to a displacement in the temperature of the loss of combined water mass toward higher temperatures.

## 3. Results

### 3.1. Study of Mechanical Strengths in Mortars

The compressive strength values for the different curing ages are summarized in Table 3.

As the obtained results show, for the 8 h curing age, the accelerator slightly improved the compressive strengths of the cement-only mortars, except for SKR, which almost doubled the value of the control mortar with no accelerator (CON). After 8 curing h, the compressive strength of the FCC + SKR mortar was almost 4-fold higher (26.03 MPa) than that of the control mortar with no accelerator (6.85 MPa) and almost twice that of the control mortar with the same accelerant (12.76 MPa). Accelerators improved the compressive strengths of both the cement-based and FCC-containing mortars for the 24 h curing age. The only exception was the mortar containing only cement with KOH as an accelerator. When the FCC was added and accelerators were used, the 24 h strength was around 50 MPa, which is a very high value for such a short curing age. The KOH began to worsen the mortars’ compressive strength after 48 h. The other two accelerators yielded higher values than the corresponding controls, which were similar to one another, with a similar trend after 28 curing days. The mortars containing FCC for the 28-day curing age yielded compressive strength values above 88 MPa, except for that containing KOH. These values are very high, which would confirm mineral addition with great pozzolanic activity.

**Figure 1:**
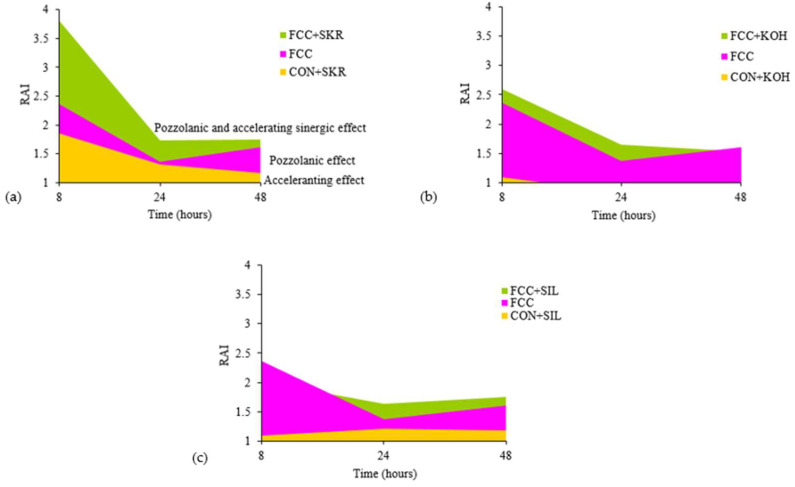
RAI values or the different accelerating additives during the 8 h to 48 h periods: (**a**) SKR accelerator; (**b**) KOH accelerator; (**c**) SIL accelerator.

To quantify the contribution of accelerating additives and FCC, the Resistance Activity Index (RAI) value was calculated as the ratio between the strength of each mortar and the control mortar with no type of accelerator or mineral addition. Figure 1 represents the values obtained by each accelerator for the 8 h to 48 h periods.

From the obtained results, it was concluded that the SKR accelerant was an additive with a synergic effect with FCC. KOH appeared to be beneficial in the mortar with FCC, but only for the first curing hours. When approaching 48 h, it had no benefit on the FCC mortar. Unlike the mortar with KOH, the mortars with SIL and those containing FCC displayed improved compressive strength from 24 curing hours.

### 3.2. Thermogravimetric Analysis in Pastes

The curing ages chosen for the thermogravimetric studies were 8, 24, and 48 h and 28 days for the pastes cured at 20 °C. The percentages of total mass loss (%T_ML_), mass loss due to the dehydroxylation of portlandite (%CH), the percentage of bound water in cementing phases (%BW) for the different tested pastes are shown in Table 4.

As seen in Table 4, the addition of accelerators influenced the quantity of portlandite for the first curing hours (8–48 h) in the control pastes and those containing FCC. For 28 curing days, the amount of portlandite was similar in all the controls and was lower in the pastes with FCC and accelerators. Despite there being a smaller amount of portlandite in the control pastes with SKR, they had a larger amount of combined water (BW) in the cementing phases, with a bigger amount of hydration products. For the silicate-based accelerator control pastes, they were slightly lower than the control paste with no accelerator. Compared to the controls, the amount of portlandite in the pastes with FCC was smaller due to the pozzolanic reaction. When using the accelerators with FCC, once again the combined water of the paste with SKR was the largest of all, which confirmed the synergistic effect of FCC and SKR.

**Figure 2:**
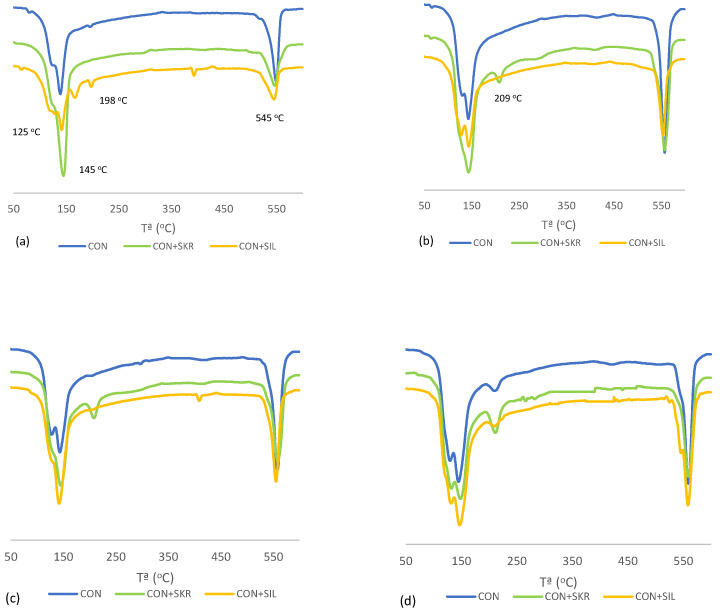
DTG curves for the control pastes at different curing ages: (**a**) 8 h; (**b**) 24 h; (**c**) 48 h; (**d**) 28 days.

**Figure 3:**
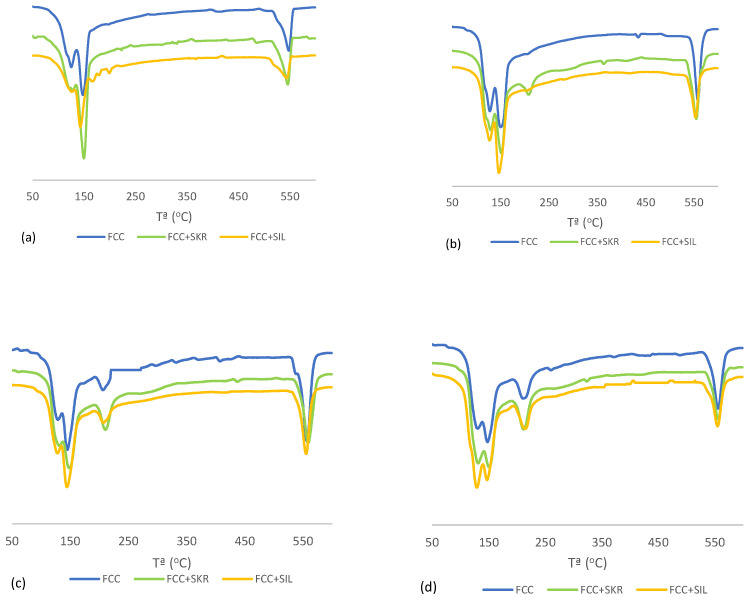
DTG curves for the FCC pastes at different curing ages: (**a**) 8 h; (**b**) 24 h; (**c**) 48 h; (**d**) 28 days.

Figure 2 and Figure 3 display the DTG (derivative thermogravimetric curves) curves of pastes. The peaks in Figure 2a are 125 °C and 145 °C, corresponding to the loss of combined water of C-S-H and ettringite; these two peaks overlap. The peaks at 167 °C and 198 °C correspond to the loss of water of the gypsum (calcium sulfate dihydrate) present in cement. Finally, the peak at 545 °C corresponds to portlandite dehydroxylation [28]. Figure 2b depicts the peak corresponding to the loss of combined water associated with C-A-S-H and C-A-H [28] (209 °C). As shown, the peak corresponding to portlandite was more visible at a longer curing age as a result of the progress undergone during the hydration reaction of cement. In the pastes that contained FCC (Figure 3), the peak that corresponded to C-A-S-H and C-A-H was the most visible for all the pastes as of 48 curing hours due to the pozzolanic reaction.

## 4. Discussion

When analyzing the results obtained in the mechanical strengths section and those in the existing literature, we observed similarities and differences. The existing literature about the use of hydroxides in cement mortars is limited. Smaoui et al. [23] have explored employing high-alkali concrete by adding NaOH pellets in the mixing water to raise the system’s alkali level to 1.25% Na_2_O by cement mass. They concluded that NaOH addition caused significant reductions in flexural and compressive strengths, which were attributed to a more reticular and porous paste. Wang et al. [29] have studied the influence of 2% and 5% NaOH on cement kiln dust (CKD)–FA binders. They observed at 24 °C that the mortars with 2% NaOH had better compressive strengths than the mortar without NaOH at all the curing ages (3, 7, 28 and 56 days). The mortars with 5% NaOH obtained better results than the control did for 3, 7, and 28 days, with the worst results seen for 56 curing days. The results herein obtained are consistent with those reported by Wang et al. [29].

Regarding a silicate-based accelerator, Coppola et al. [30] have applied sodium silicate to manufacture fiber-reinforced shotcrete manufactured with different fibers. The concretes with sodium silicate achieved compressive strengths that fell within the 50–85% range, which was higher than the concretes without sodium silicate at 1 curing day. However, at 28 curing days, the compressive strengths of the concretes with this additive were 35–45% less than their respective controls. Our research detected no decrease in compressive strength when using sodium silicate at 28 curing days.

The SKR safety data sheet, which is accessible at [https://www.buildsite.com/pdf/sika/Sika-Rapid-1-SDS-2084451.pdf, accessed on 2 February 2024], states that it contains sodium nitrate (10–20%), sodium thiocyanate (5–10%) and 2,2′-methylminodiethanol (1–5%). The last component has no influence on compressive strength, with benefits only for protecting reinforcement against corrosion [31]. On sodium nitrate use, Xu et al. [31] have established that sodium and calcium nitrates accelerate the hydration reaction of C_3_A. Nitrate participates in the hydration reaction by forming Ca_4_Al_2_[OH]_12_[NO_3_]_2_.xH_2_. They are effective in achieving higher initial strengths in concrete. Wise et al. [32] have concluded that thiocyanates accelerate the hydration rate of PC cured at ambient and low temperatures. SKR was the most effective accelerator in the present research work. It is unknown which of the accelerator components is the most effective because we are unaware of their exact composition, but we found that it is effective at both the first curing ages and at 28 curing days.

When comparing the results obtained in the thermogravimetric analysis section to the existing literature, similarities are observed. For example, Hoang et al. [4] have used a mixture of sodium thiocyanate, diethanolamine, and glycerol in blended cement with FA. They concluded that the paste with the accelerator had bigger quantities of AFm phases, which denoted a higher degree of hydration. In the pastes with accelerators, the fact that the amount of portlandite was smaller at short curing ages could be due to partial C_3_S hydration inhibition as a result of a greater formation of AFm phases, which favor these accelerators. This is why the amount of bound water was maintained, or even increased, as C-S-H did not form but other hydration products did, which made the amount of combined water larger [33]. When employing SIL, the quantities of portlandite and bound water were the smallest of all the pastes. Other authors have found similar results. Shi et al. [34] have observed a smaller amount of bound water and a lower degree of hydration at short curing ages in cement pastes containing SIL. However, these values were compensated at longer ages. In fact, the reported peaks herein, which were due to water loss from gypsum, were observed only in the control paste with SIL at 8 curing hours. This fact shows that cement hydration with SIL during the first curing hours was slower than the control and SKR pastes.

## 5. Conclusions

From the obtained results, it can be concluded that:

- The SKR commercial accelerator is the additive with the best behavior, especially during the first 8 curing hours, and no reduction in strength takes place for long curing ages (28 days). This indicates good compatibility between FCC and SKR.
- KOH appears beneficial in the mortar with FCC, but only for the first curing hours. When approaching 48 h, it exerts no benefit on the FCC mortar.
- Unlike the mortar with KOH, the mortars with SIL containing FCC displayed improved compressive strength from 24 curing hours.
- The mortars containing FCC for the 28-day curing age obtained higher compressive strength values than 88 MPa, except that containing KOH. These values confirm that FCC is an SCM with excellent pozzolanic activity.
- Accelerators influence cement hydration by favoring the formation of Afm phases at early curing ages.

## Figures and Tables

**Table 1:** Chemical compositions of cement and FCC (%).

	SiO_2_	Al_2_O_3_	Fe_2_O_3_	CaO	MgO	SO_3_	Na_2_O + K_2_O	P_2_O_5_	TiO_2_	LOI *
CEM	19.29	5.22	3.51	61.75	2.07	3.55	1.23	0.26	0.27	1.96
FCC	47.76	49.25	0.60	0.11	0.17	0.03	0.33	0.02	1.22	0.51

* LOI: Loss on ignition.

**Table 2:** Mortar doses.

	Cem (g)	Sand (g)	FCC (g)	Accel. (g)	H_2_O (g)	Superplast. (g)	Work. (mm)
CON	450.0	1350.0	_	_	202.5	_	110
FCC	450.0	1215.0	135.0 *	_	202.5	3.2	110
CON + KOH	450.0	1350.0	_	9.0	202.5	1.1	111
FCC + KOH	450.0	1215.0	135.0 *	9.0	202.5	6.2	119
CON + SIL	450.0	1350.0	_	9.0	202.5	1.2	112
FCC + SIL	450.0	1215.0	135.0 *	9.0	202.5	5.7	113
CON + SKR	450.0	1350.0	_	9.0	202.5	_	113
FCC + SKR	450.0	1215.0	135.0 *	9.0	202.5	3.2	116

* Sand was replaced with 10% FCC by mass.

**Table 3:** Compressive strengths (MPa) of mortars at different curing ages.

	8 h	24 h	48 h	28 d
CON	6.85 ± 0.51	29.23 ± 1.17	34.47 ± 2.06	56.96 ± 1.31
FCC	16.18 ± 1.24	39.93 ± 2.15	55.44 ± 2.23	88.54 ± 2.61
CON + KOH	7.51 ± 0.82	24.60 ± 1.76	28.86 ± 0.45	48.13 ± 0.86
FCC + KOH	17.86 ± 1.05	48.13 ± 1.25	52.98 ± 1.75	68.10 ± 2.05
CON + SIL	7.56 ± 0.79	35.42 ± 2.03	40.69 ± 0.35	61.25 ± 1.67
FCC + SIL	13.83 ± 0.85	47.72 ± 0.52	60.38 ± 2.10	89.30 ± 1.92
CON + SKR	12.76 ± 1.32	38.67 ± 1.25	40.40 ± 0.97	64.16 ± 1.23
FCC + SKR	26.03 ± 0.65	50.61 ± 0.79	60.43 ± 1.56	91.60 ± 1.46

**Table 4:** Percentages of total mass loss (T_ML_), and mass loss due to dehydroxylation of portlandite (CH) and bound water (BW).

	8 h			24 h			48 h			28 d		
	T_ML_	CH	BW	T_ML_	CH	BW	T_ML_	CH	BW	T_ML_	CH	BW
CON	6.8	1.0	5.8	13.2	2.3	10.9	15.9	2.7	13.2	21.1	3.4	17.7
CON + SIL	5.4	0.7	4.7	9.6	2.2	7.4	13.8	2.0	11.8	20.2	3.5	16.7
CON + SKR	8.1	0.8	7.3	15.6	1.3	14.3	16.9	2.4	14.5	22.4	3.4	19.0
FCC	7.8	0.9	6.9	13.5	1.2	12.3	15.7	2.2	13.5	23.5	2.3	21.2
FCC + SIL	5.9	0.5	5.4	11.6	1.1	10.5	14.6	1.6	13.0	22.8	1.8	21.0
FCC + SKR	8.6	1.0	7.6	14.9	1.4	13.5	17.6	1.8	15.8	25.1	1.8	23.3

## Data Availability

Data will be made available on request.
